# Cross-sectional study of individual and environmental factors associated with life-space mobility among community-dwelling independent older people

**DOI:** 10.1186/s12199-021-00936-2

**Published:** 2021-01-18

**Authors:** Tomoha Miyashita, Etsuko Tadaka, Azusa Arimoto

**Affiliations:** 1 Health and Welfare Center, Totsuka Ward Office, 16-17 Totsukacho, Totsuka-ku, Yokohama, 244-0003 Japan; 2 Department of Community Health Nursing, Graduate School of Medicine, Yokohama City University, 3-9 Fukuura, Kanazawa-ku, Yokohama, 236-0004 Japan

**Keywords:** Community, Cross-sectional study, Environment, Individuality, Independent older people, Life, Mobility, Primary prevention

## Abstract

**Background:**

Life-space mobility is reflected in comprehensive longevity and health outcomes and is also an important indicator for preventing mortality and decline in well-being among older people. However, a comprehensive framework of life-space mobility and modifiable individual and environmental factors has not been well validated among community-dwelling independent older people, for primary prevention. We examined individual and environmental factors affecting life-space mobility among community-dwelling independent older people.

**Methods:**

This cross-sectional study included 3500 community-dwelling independent older people randomly selected using the National Basic Resident Registration System in Japan. Life-space mobility was measured using the Japanese version of the Life-Space Assessment (LSA) instrument, which is used to assess an individual’s pattern of mobility. Negative multivariate binomial regression analysis was performed in a final sample of 1258 people. Individual factors (including physical, mental, and social characteristics) and environmental factors (including the social and material environment) were measured and analyzed as potential factors.

**Results:**

Negative multivariable binomial regression analysis, adjusted for demographics, showed that LSA score was associated with locomotive syndrome (*β* = − 0.48, 95% confidence interval [CI] = − 0.24 to − 0.73), depression (*β* = − 0.29, 95% CI = − 0.03 to − 0.55), health literacy (*β* = 0.20, 95% CI = 0.39–0.01), and participation in community activities (*β* = 0.23, 95% CI = 0.03–0.43) among individual factors, and receipt of social support (*β* = − 0.19, 95% CI = 0.00 to − 0.38) and social network (*β* = 0.29, 95% CI = 0.48–0.10) among environmental factors.

**Conclusions:**

Our findings suggest that modifiable individual factors and environmental factors are related to life-space mobility among community-dwelling older people.

## Background

Life-space mobility is defined as the spatial area within which a person travels over a specified period in daily life, and considers not only the extent of movement but also the frequency of movement and any assistance needed [1]. Restricted life-space mobility can reduce participation in social activities, which can lead to low utilization of available community amenities [2], poor quality of life (QOL) [3], and increased mortality [4–6]. In contrast, enhanced life-space mobility provides individuals with a greater variety of daily activities and improved well-being [7]. Therefore, life-space mobility is reflected in comprehensive longevity and health outcomes and is also an important indicator for preventing mortality and declines in well-being among older people. The prevention of decline and maintaining or expanding of life-space mobility among community-dwelling independent older adults is important for primary prevention to limit the onset of restricted life-space mobility and maximize healthy longevity.

Previous studies among healthy, independent, community-dwelling older people have shown that life-space mobility, including related indicators such as frequencies of going outside and being homebound, is associated with various individual and environmental factors. Individual factors include sex [8–10], age [11–13], ethnicity [8], physical function [10, 14–18], cognition [15, 17, 19], activities of daily living [9], mobility [12, 16, 18], depressive symptoms and mental health [15, 20, 21], physical activity and physical performance [12, 14, 22–24], lifestyle [12, 15], and diseases and symptoms such as pain or fatigue [14, 15, 18]. Environmental factors include perceived entrance-related barriers [25], access to social services [26], social support and social assistance [11, 12, 14, 15], neighborhood green space [27], and environmental facilitators [28].

The above evidence suggests that various individual factors and environmental factors may play an important role in life-space mobility among community-dwelling independent older people. However, the above studies have the following limitations. First, representativeness of the study participants as community-dwelling independent older people was unclear because the criteria of community-dwelling independent older people was age 65 years and over, and whether a participant was independent (not certified as needing long-term care/support) was not sufficiently validated. For the prevention of decline and for maintaining or expanding of life-space mobility in older people, primary prevention among community-dwelling independent older adults is urgently needed. Second, individual and environmental factors include non-modifiable factors, and they have been investigated separately. Life-space assessment is a concept that takes into account the interaction between individuals and their environment [29, 30]. Especially in Japan, where the aging of the population is expected to accelerate in the future, to facilitate individual and population-level behavior change among community-dwelling independent older people, it is necessary to broadly clarify the comprehensive frameworks for identifying modifiable factors of the life-space mobility. Regarding comprehensive modifiable individual and environmental factors for life-space mobility and modifiable individual and environmental factors, in addition to previously reported factors, health literacy related to subjective mobility disability [31] and community activities, such as group activities that promote frequency of going outside [16, 17], are needed. Also, the social network of family and friends [32] and perceived neighborhood environment are important [27, 33] because effective population-based approaches for the prevention of decline and for maintaining or expanding of life-space mobility that have a long-term impact on community-dwelling independent older people can be expected with interventions using comprehensive modifiable individual and environmental factors.

The purpose of the present study was to examine individual and environmental factors affecting life-space mobility among community-dwelling independent older people. Japan is a “super-aging” society, and the importance of preventive strategies will increase ahead of the world in the future. This study contributes to our understanding of factors that affect independent life-space mobility among community-dwelling older adults and can help to guide the design of enhanced preventive strategies to improve life-space mobility in super-aged societies. In the context of the present study, individual factors were defined as modifiable physical, mental, and social characteristics among independent older people. Environmental factors were defined as modifiable social and physical (meaning “material”) environment where people live and conduct their daily lives, which are common to independent older people and can be modified through intervention [34].

## Methods

### Participants

The criteria for including participants in this study were as follows: (1) being age 65 years and over; (2) being independent older people; and (3) living in the community. The criteria for being an independent older person was identified according to the Certified Level of Need for Long-Term Care Insurance in Japan (*Kaigo Hoken* in Japanese); individuals who were not certified as needing long-term care/support were considered independent older people. To ensure representativeness of community-dwelling independent older people, 3500 individuals were randomly selected among “community-dwelling” “independent” “older” people certified in Yokohama City, the largest designated city in Japan according to the National Basic Resident Registration System administered by the Ministry of Internal Affairs and Communications of Japan. Self-administered questionnaires were mailed to 3500 people, among which 19 questionnaires were undeliverable owing to an invalid address. As a result, 3481 questionnaires were distributed, and a total of 1730 people responded (response rate 49.7%). Of these, a total of 472 people was excluded based on the exclusion criteria: (1) certified need for long-term care/support need, (2) under 65 years old or unknown age, (3) unknown sex, (4) unknown life-space assessment (LSA). Thus, 1258 older adults comprised the final sample in the analysis (valid response rate 36.1%). The data collection period was October 24 to November 5, 2017.

### Measurements

#### Dependent variable

Life-space mobility was measured using the Japanese version of the Life-Space Assessment (LSA) [35], which is used to assess an individual’s pattern of mobility across five levels of their life space (from within the home to outside of their town), during the month prior to assessment. For each life-space level, participants were asked how often they moved or traveled within a given area (less than once a week, 1–3 times each week, 4–6 times each week, or daily) and whether they required assistance from another person or an assistive device (“yes” or “no”). LSA scores range from 0 (restricted to one’s bedroom) to 120 (traveling outside of one’s town daily without assistance), with higher scores indicating greater life-space mobility [1, 35]. This instrument has been previously validated [1, 13, 35].

#### Independent variables

Demographic characteristics included age, sex, marital status (married/divorced/widowed/unmarried), household composition (living with a spouse/with a spouse and children/alone/with children/with children and grandchildren/other), job status (employed/unemployed), and whether respondents were currently under treatment for any diseases, confirmed using the Health Statistics of the Number of Individuals Receiving Medical Treatment for Older People in Japan (yes/no).

We considered a comprehensive set of individual factors as follows. Physical function included visual and hearing function (only visual impairment/only hearing impairment/both/none), urinary incontinence (frequent/sometimes/never), and history of falls (yes/no). Those who responded that they had a visual or hearing impairment were treated as having a visual or hearing impairment. Participants who reported that urinary incontinence was frequent or occasional were considered to have urinary incontinence. Locomotion was measured using the Short Form of the Geriatric Locomotive Function Scale (GLFS-5). Scores range from 0 to 20, with higher scores indicating a higher risk of locomotive dysfunction [36]. This instrument has been previously validated [36, 37] Cognitive function was measured using the self-administered dementia checklist (SDC). Scores range from 10 to 40, with higher scores indicating a higher risk of cognitive function decline [38]. This instrument has been previously validated [38]. Depression was measured using the Geriatric Depression Scale, five-item version (GDS-5). Scores range from 0 to 5, with higher scores indicating higher depression risk [39]. This instrument has been previously validated [39, 40]. Health literacy was measured using the Communicative Critical Health Literacy Scale (CCHL), which assesses the cognitive and social skills that determine the motivation and ability of individuals to gain access to, understand, and use information in ways that promote and maintain good health. Scores range from 5 to 25, with higher scores indicating higher health literacy [41]. This instrument has been previously validated [41]. Participation in community activities was measured by asking participants whether they engaged in community activities, with a yes/no response.

Environmental factors were as follows. The exchange of social support was measured using the Social Support Exchange Scale (SSES), which assesses the receiving and giving of support. Scores range from 12 to 48, with higher scores indicating higher degrees of receiving and giving social support [42]. This instrument has been previously validated [42]. Social networks were measured using the Lubben Social Network Scale, Japanese version (LSNS-6), which measures the size, closeness, and frequency of contact with each respondent’s social network of family and friends. Scores range from 0 to 30, with higher scores indicating a greater social network of family and friendships [32]. This instrument has been previously validated [32]. Perceived neighborhood environment was measured using the Abbreviated Neighborhood Environment Walkability Scale, Japanese version (ANEWS-J), which measures proximity to neighborhood land uses, such as restaurants and retail stores (land-use mixed access), neighborhood sidewalks/bike lanes, and neighborhood esthetics. According to Cerin et al., the author of the original ANEWS [43], patterns of association between environmental characteristics may vary across urban and other (e.g., rural) areas, but may form a single pattern with the same environmental characteristics (e.g., high street connectivity predictive of high land-use mix). This study area was one urban city (Yokohama City, in Japan), which has the same environmental characteristics in the three attributes of the eight categories of ANEWS (residential density, land-use mix diversity, and street connectivity), and they may form a single pattern, not vary across respondents. Also, according to Cerin et al. [43], the two attributes of traffic safety and crime safety can depend on trait anxiety in respondents. Based on the above and previous references [26, 28, 33], we considered that three categories could help to guide the design of key preventive strategies for maintaining or expanding life-space mobility among community-dwelling independent older people. Scores range from 4 to 6, with higher scores indicating a more favorable neighborhood environment [44]. This instrument has been previously validated [44].

### Statistical analysis

Univariate analysis using Spearman’s correlation was conducted to examine the correlations between LSA scores and independent variables. Given that LSA scores were assumed to have a highly skewed distribution and there is currently no established cutoff point among community-dwelling older people, negative multivariable binomial regression was used to investigate whether each significant independent variable was related to the LSA score, which was divided into two categories according to mean score. Life-space mobility in the group with a high LSA score refers to an area within and outside of one’s town. Life-space mobility in the group with a low LSA score refers to an area limited to one’s home and neighborhood. In negative multivariable binomial regression analysis, adjustment for covariates was conducted for age, sex, and job status. Furthermore, generalized linear models were conducted to confirm robustness of the study results. The results are expressed as standardized partial regression coefficient (*β*) with 95% confidence intervals (CIs). The results were regarded as statistically significant with *p* < 0.05 or if the 95% CI did not include 1. IBM SPSS software, version 22 (IBM Corp., Armonk, NY, USA) was used for the analyses.

## Results

Table 1 shows the demographic characteristics of participants. Respondents’ mean age was 73.5 years (SD = 6.1 years, range 65–100), 60.7% were 65–74 years old, 50.1% were male, 76.7% were married, 47.2% lived with a spouse, and 75.4% were unemployed.

**Table 1:** Demographic characteristics of study participants (*n* = 1258)

Characteristics	*n*, mean ± SD	% (range)
Age		
All	73.5 ± 6.1	(65–100)
65–74 years	764	60.7
75–84 years	427	33.9
85 years and older	66	5.3
Sex		
Male	630	50.1
Female	628	49.8
Marital status		
Married	965	76.7
Divorced/widowed	247	19.6
Unmarried	41	3.3
No response	5	0.4
Household composition		
Living with spouse	594	47.2
Living with spouse and children	255	20.3
Living alone	187	14.9
Living with children	110	8.7
Living with children and grandchildren	56	4.5
Other	53	4.2
No response	3	0.2
Job status		
Employed	295	23.4
Unemployed	948	75.4
No response	15	1.2
Diseases currently under treatment		
Yes	962	76.5
High blood pressure	505	40.1
Diabetes/hyperlipidemia	284	22.6
Arthritis/osteoporosis	162	12.9
Cataract/glaucoma	156	12.4
Myocardial infarction/angina	93	7.4
Cancer	72	5.7
Pneumonia/bronchitis	56	4.5
Stroke/cerebral infarction	43	3.4
Mental disorders	15	1.2
Others	194	15.4

*SD* standard deviation

Table 2 shows the LSA scores, as well as individual and environmental characteristics of participants. The median LSA score was 92.0 (interquartile range [IQR] 78–102). In terms of vision and hearing function, 76.2% of participants had good vision and hearing levels. The mean GLFS-5 score was 2.0 (SD 3.0), and the mean SSES score was 18.9 (SD 5.3).

**Table 2:** LSA and individual and environmental characteristics of study participants (*n* = 1258)

			*N* or mean (median)	% or SD (IQR/range)
Life-space mobility	LSA points		91.2 (92.0)	20.7 (78.0–102.0)
Individual factors	Hearing impairment		138	11.0
	Visual impairment		69	5.5
	Visual and hearing impairment		53	4.2
	No response		50	3.5
	Urinary incontinence	Frequent	13	1.0
		Sometimes	247	19.6
		Never	994	79.0
		No response	4	0.3
	Fall in the past year	No	1050	83.5
		Yes	205	16.3
		No response	3	0.2
	GLFS-5 points		2.0	3.0 (0–20)
	SDC points		12.2	2.6 (10–33)
	GDS-5 points		0.8	1.1 (0–5)
	CCHL points		19.1	4.5 (5–25)
	Participation in community activity	Yes	617	49.0
Environmental factors	SSES points	Receiving	8.1	2.6 (6–21)
		Giving	10.8	3.8 (6–24)
	LSNS-6 points	Family	8.2	3.2 (0–15)
		Friendships	6.8	4.0 (0–15)
	ANEWS-J points	Land use mix-access	3.1	0.5 (1.5–4.0)
		Sidewalk/bike lanes	2.4	0.8 (1.0–4.0)
		Esthetics	2.5	0.8 (1.0–4.0)

*LSA* Life-Space Assessment, *GLFS-5* Geriatric Locomotive Function Scale, *CCHL* Communicative Critical Health Literacy Scale, *SDC* self-administered dementia checklist, *GDS-5* Geriatric Depression Scale, *SSES* Social Support Exchange Scale, *LSNS-6* Lubben Social Network Scale, *ANEWS-J* Abbreviated Neighborhood Environment Walkability Scale, Japanese version, *SD* standard deviation, *IQR* interquartile range

There were significant correlations among demographic characteristics, individual and environmental factors, and life-space mobility for 19 factors using Spearman’s correlation coefficient. The demographic characteristics showing significant correlations with life-space mobility were age (*ρ* = − 0.176, *p* < 0.01), sex (*ρ* = 0.110, *p* < 0.01), and two other factors. Individual factors showing significant correlations with life-space mobility were GLFS-5 (*ρ* = − 0.313, *p* < 0.01), SDC (*ρ* = − 0.183, *p* < 0.01), and seven other factors. Environmental factors showing significant correlations with life-space mobility were SSES receiving (*ρ* = 0.155, *p* < 0.01), SSES giving (*ρ* = 0.061, *p* < 0.1), and four other factors. Considering multicollinearity, 16 of the 19 factors were used as independent variables, and age, sex, and job status were entered into the multivariate logistic regression analysis as control variables.

Table 3 shows the age-, sex-, and job status-adjusted associations between life-space mobility and the independent variables in negative multivariable binomial regression analysis. As continuous variables showed a non-normal distribution, these were divided into two groups according to the median value. Of the 16 identified factors, 6 were associated with life-space mobility: GLFS-5 (*β* = − 0.48, 95% CI = − 0.24 to − 0.73), GDS-5 (*β* = − 0.29, 95% CI = − 0.03 to − 0.55), CCHL (*β* = 0.20, 95% CI = 0.39–0.01), participation in community activities (*β* = 0.23, 95% CI = 0.03–0.43), SSES receiving (*β* = − 0.19, 95% CI = 0.00 to − 0.38), and LSNS-6 friendships (*β* = 0.29, 95% CI = 0.48–0.10).

**Table 3:** Demographic, individual, and environmental factors associated with life-space mobility in negative multivariable binomial regression analysis

		*β*	95% Cl		
			LL	UL	
Demographic characteristics	Diseases currently under treatment	0.02	− 0.21	0.25	n.s.
Individual factors	Visual impairment	0.24	− 0.10	0.58	n.s.
	Hearing impairment	0.11	− 0.16	0.38	n.s.
	Urinary incontinence	0.09	− 0.15	0.33	n.s.
	Fall experience in the past year	0.05	− 0.21	0.31	n.s.
	GLFS-5	− 0.48	− 0.24	− 0.73	
	SDC	0.12	− 0.07	0.31	n.s.
	GDS-5	− 0.29	− 0.03	− 0.55	
	CCHL	0.20	0.39	0.01	
	Participation in community activities	0.23	0.03	0.43	
Environmental factors	SSES receiving	− 0.19	0.00	− 0.38	
	SSES giving	− 0.07	− 0.26	0.12	n.s.
	LSNS-6 family	− 0.16	− 0.34	0.03	n.s.
	LSNS-6 friendships	0.29	0.48	0.10	
	ANEWS-J land use mix-access	− 0.11	− 0.31	0.08	n.s.
	ANEWS-J esthetics	− 0.12	− 0.30	0.07	n.s.

Negative multivariable binomial regression analysis, with adjustment for age, sex, and job status. Diseases currently under treatment yes = 1, no = 0; with visual or hearing impairment = 1, without = 0; with urinary incontinence = 1, without = 0; with a fall experienced in the past year = 1, without = 0; with higher risk of locomotive dysfunction (GLFS-5), cognitive function decline (SDC), or depression (GDS-5) = 1, without = 0; high health literacy (CCHL) = 1, or not = 0; high participation in community activities = 1, or not = 0; high SSES receiving and giving, high LSNS-6 family and friendships, and high ANEWS-J land use mix-access and esthetics = 1 or not = 0

*β* standardized partial regression coefficient, *Cl* confidence interval, *LL* lower limit, *UL* upper limit, *n.s.* not significant, *GLFS-5* Geriatric Locomotive Function Scale, *CCHL* Communicative Critical Health Literacy Scale, *SDC* self-administered dementia checklist, *GDS-5* Geriatric Depression Scale, *SSES* Social Support Exchange Scale, *LSNS-6* Lubben Social Network Scale, *ANEWS-J* Abbreviated Neighborhood Environment Walkability Scale, Japanese version

Table 4 shows the results of generalized linear models, after adjusting for the age, sex, and baseline value, showed that GLFS-5 (*β* = − 7.03, 95% CI = − 9.86 to − 4.21), GDS-5 (*β* = − 5.28, 95% CI = − 8.27 to − 2.29), CCHL (*β* = 2.98, 95% CI = 0.59–5.37), participation in community activities (*β* = 3.37, 95% CI = 0.98–5.76), SSES receiving (*β* = − 4.21, 95% CI = − 1.80 to − 6.62), and LSNS-6 friendship (*β* = 3.88, 95% CI = 1.42–6.33) were associated with life-space mobility, consistent with the results of negative binomial regression.

**Table 4:** Demographic, individual, and environmental factors associated with life-space mobility in generalized liner model

	*β*	95% Cl	
		LL	UL
Individual factors			
GLFS-5	− 7.03	− 9.86	− 4.21
GDS-5	− 5.28	− 8.27	− 2.29
CCHL	2.98	0.59	5.37
Participation in community activities	3.37	0.98	5.76
Environmental factors			
SSES receiving	− 4.21	− 1.80	− 6.62
LSNS-6 friendships	3.88	1.42	6.33

Generalized linear model, with adjustment for age, sex, and job status. With higher risk of locomotive dysfunction (GLFS-5), or depression (GDS-5) = 1, without = 0; high health literacy (CCHL) = 1, or not = 0; high participation in community activities = 1 or not = 0; high SSES receiving and giving, high LSNS-6 family and friendships

*β* standardized partial regression coefficient, *Cl* confidence interval, *LL* lower limit, *UL* upper limit, *n.s.* not significant, *GLFS-5* Geriatric Locomotive Function Scale, *CCHL* Communicative Critical Health Literacy Scale, *GDS-5* Geriatric Depression Scale, *SSES* Social Support Exchange Scale, *LSNS-6* Lubben Social Network Scale

## Discussion

The importance of this study lies in its contribution to clarifying the association between life-space mobility and the examined comprehensive set of modifiable individual factors and environmental factors among a representative sample of community-dwelling independent older people. There are two new findings in this study, in addition to those of previous studies. First, in terms of individual factors, locomotive function (GLFS-5), depression (GDS-5), communicative critical health literacy (CCHL), and participation in community activities were significantly associated with life-space mobility after adjusting for age, sex, and job status. Additionally, life-space mobility might be mutually related to individual physical, mental, and social factors. It is evident from previous studies that physical function and symptoms are related to life-space mobility among community-dwelling older adults [1, 36]. Moreover, older people with higher locomotion as well as physical and mental function demonstrate higher levels of walking [45]. Such older people have good cognitive functioning outside of their home [46] and actively engage with the community, thus expanding their life space. Health literacy, as identified in this study, refers to advanced skills of participation in everyday activities and using health information to exert greater control over life events [41]. Older people with higher health literacy may have better access to various resources, greater interaction with their neighbors, and may participate in social activities to expand their life-space mobility.

Second, in terms of environmental factors, the presence of a social network of friendships and receiving social support was significantly associated with life-space mobility after adjusting for age, sex, and job status. A previous study showed that those who reported greater social involvement tended to have larger life spaces [17]. The neighborhood context impacts the development of social networks and can generate community commitment, such as a sense of attachment and cohesion among community residents [47]. It is conceivable that older people with large social networks have high community commitment and stronger ties with the community. Life space will be expanded because there are many opportunities to go out into the community to engage with friends. Previous research has also pointed out the relationship with social support [14, 15]. However, by focusing on giving and receiving social support, it is possible to clarify the kinds of social support that are important. In this study, life-space mobility and receiving social support showed a negative association. Because the study participants were independent older adults who did not need assistance, it is thought that our result was because they have few opportunities for assistance. Although older people tend to be regarded as the recipients of support, it is necessary to create communities that respect the autonomy of older residents and their capacity for self-help and to support one another, regardless of age. Saajanaho et al. [48] showed that supporting older people as they strive for relevant personal goals in their lives might contribute to enlarging their life space and thereby improving quality of life at older ages. Social networks and social support may serve to provide mutual help in moving toward goals of active living, and to increase abilities and opportunities to expand life-space mobility among community-dwelling older people. That is, life-space mobility might be mutually related to environmental factors in maximizing individual abilities and opportunities.

This study has several limitations. First, owing to the cross-sectional design, it was impossible to establish causal relationships between life-space mobility and individual and environmental factors. Second, the target population was limited to one urban area of Yokohama, Japan. It is unclear whether the results in Yokohama City can be generalized to suburban and rural areas; therefore, further study is needed.

## Conclusions

Our findings suggest that the modifiable individual factors and environmental factors examined in this study are related to life-space mobility in community-dwelling older people. The development of an intervention model that takes into account comprehensive individual and environmental factors is needed to prevent decline and to maintain or expand life-space mobility among community-dwelling independent older people. Future longitudinal studies are needed to validate our findings.

## Data Availability

The datasets generated and analyzed during the current study are not publicly available because we did not receive consents for data provision to the third party.

## Abbreviations

ANEWS-J: Abbreviated Neighborhood Environment Walkability Scale, Japanese version
β: Standardized partial regression coefficient
CCHL: Communicative Critical Health Literacy Scale
Cl: Confidence interval
LL: Lower limit
UL: Upper limit
GDS-5: Geriatric Depression Scale
GLFS-5: Geriatric Locomotive Function Scale
IQR: Interquartile range
LSA: Life-Space Assessment
LSNS-6: Lubben Social Network Scale
SD: Standard deviation
SDC: Self-administered dementia checklist
SSES: Social Support Exchange Scale
